# Pathogenesis of Osteoarthritis: Risk Factors, Regulatory Pathways in Chondrocytes, and Experimental Models

**DOI:** 10.3390/biology9080194

**Published:** 2020-07-29

**Authors:** Yuchen He, Zhong Li, Peter G. Alexander, Brian D. Ocasio-Nieves, Lauren Yocum, Hang Lin, Rocky S. Tuan

**Affiliations:** 1 Center for Cellular and Molecular Engineering, Department of Orthopaedic Surgery, University of Pittsburgh School of Medicine, Pittsburgh, PA 15219, USA; yuche@pitt.edu (Y.H.); alanzhongli@pitt.edu (Z.L.); pea9@pitt.edu (P.G.A.); BDO12@pitt.edu (B.D.O.-N.); LAY23@pitt.edu (L.Y.); 2 McGowan Institute for Regenerative Medicine, University of Pittsburgh School of Medicine, Pittsburgh, PA 15219, USA; 3 Department of Bioengineering, University of Pittsburgh Swanson School of Engineering, Pittsburgh, PA 15219, USA; 4 Institute for Tissue Engineering and Regenerative Medicine, The Chinese University of Hong Kong, Hong Kong, China

**Keywords:** osteoarthritis, pathogenesis, experimental model, disease modifying osteoarthritis drugs, microphysiological systems

## Abstract

As the most common chronic degenerative joint disease, osteoarthritis (OA) is the leading cause of pain and physical disability, affecting millions of people worldwide. Mainly characterized by articular cartilage degradation, osteophyte formation, subchondral bone remodeling, and synovial inflammation, OA is a heterogeneous disease that impacts all component tissues of the articular joint organ. Pathological changes, and thus symptoms, vary from person to person, underscoring the critical need of personalized therapies. However, there has only been limited progress towards the prevention and treatment of OA, and there are no approved effective disease-modifying osteoarthritis drugs (DMOADs). Conventional treatments, including non-steroidal anti-inflammatory drugs (NSAIDs) and physical therapy, are still the major remedies to manage the symptoms until the need for total joint replacement. In this review, we provide an update of the known OA risk factors and relevant mechanisms of action. In addition, given that the lack of biologically relevant models to recapitulate human OA pathogenesis represents one of the major roadblocks in developing DMOADs, we discuss current in vivo and in vitro experimental OA models, with special emphasis on recent development and application potential of human cell-derived microphysiological tissue chip platforms.

## 1. Introduction

Existing since ancient times and officially named and defined in the 19th century [1], osteoarthritis (OA) has been the most common degenerative joint disease. According to data collected by US Centers for Disease Control and Prevention, OA affects 52.5 million people in the United States in 2012 and the number is expected to go up to 78 million (26%) by 2040 [2]. In the 2010 Global Burden of Disease report, OA was the 11th highest global disability contributor out of 291 health conditions studied [3], affecting both physical and mental health and with substantial healthcare costs [4]. Data from the National Institutes of Health Osteoarthritis Initiative (OAI) study demonstrated that people with multi-site, hip, or knee OA have greater odds of developing depression-related symptoms as compared to people without OA [5]. Greater suicidal ideation odds and memory loss, partially mediated by sleep and mood impairments caused by joint symptoms, are found in OA patients [6,7]. Commonly affected joints are large weight-bearing joints, such as the knee and hip [8], that are characterized by synovial distension and inflammation, thin and rough articular cartilage, and reactive bone hyperplasia at the joint edge and beneath the cartilage [9]. Radiographically, OA presents with joint space narrowing, osteophytosis, subchondral sclerosis, cyst formation, and abnormalities of bone contour [10,11]. These changes cause pain, stiffness, tenderness, and loss of mobility that often arise near the end of disease progression, greatly impacting patients’ life quality and even leading to mortality [12]. Current OA prevalence is ~15% and is predicted to reach 35% by 2030, making it the single greatest cause of disability globally. Although it remains unclear why the prevalence of OA is rising, the most likely factor is the increase of obese and aged populations [13].

OA is characterized as a failure of the joint organ that affects all the tissues in and around the joint, these affects include degradation of the articular cartilage; thickening of the subchondral bone; osteophyte formation; variable degrees of synovial inflammation; degeneration of ligaments; hypertrophy of the joint capsule; and changes in periarticular muscles, nerves, bursa, and local fat pads [14]. Among these, cartilage degradation is considered to be the central feature [15]. This is because articular cartilages are anatomically at the frontline to respond to the local biomechanical environmental, specifically absorbing and distributing mechanical loads applied to the articular joint and providing a low friction system to enable mobility. Highly regulated anabolic and catabolic mechanisms maintain and adapt cartilage to disruptive factors [16]. In OA, dysregulation caused by the presence of various biofactors leads to the loss of cartilage homeostasis, resulting in degradation of the collagen- and proteoglycan-rich extracellular matrix (ECM), fibrillation and erosion of the articular surface, cell death, matrix calcification, and vascular invasion [17].

Despite the disease being known for centuries, the exact pathogenic mechanisms of OA remain unclear. Initially, OA was considered an unavoidable age-related disease caused by biomechanical factors, i.e., “wear-and-tear”, and an imbalance in the homeostatic biochemical mechanisms of cartilage, distinct from immunologically-mediated rheumatoid arthritis [18]. However, detailed examination revealed patient-specific variability in the clinical presentation and disease progression [19]. Most cases of OA have a clear predisposing condition, such as genetics, trauma, aging, or obesity, leading to the idea that OA describes a common endpoint with different etiologies. In any case, it is now widely accepted that OA is a dynamic and complex process, involving inflammatory, mechanical, and metabolic factors that result in the inability of the articular surface to serve its function of absorbing and distributing the mechanical load through the joint that ultimately leads to joint destruction [11]. Furthermore, it is now recognized that the disease is not restricted to the cartilage or subchondral bone; rather, it results from interplay among tissues of the osteochondral complex, including adipose and synovial tissue, as well as the ligaments, tendon, and muscles that surround the joint [14]. The exact pathogenic mechanism(s) of OA are still unknown, despite modern advances in analysis and diagnosis [20], which accounts for the pre-clinical and clinical failure of a number of potential disease-modifying pharmacological therapies [21].

Articular cartilage has a relatively simple tissue composition of only a single cell type, chondrocytes, encased in an abundant ECM, in the absence of blood vessels, nerves, or lymphoid tissue. Any change in its components affects cartilage homeostasis. As the cartilage ECM is produced by chondrocytes, OA pathogenesis is therefore frequently linked to changes in chondrocyte activities, including proliferation, matrix deposition, inflammatory cytokine production, and response to signaling molecules. These changes drive cartilage degradation and therefore represent candidate therapeutic targets to reverse OA and maintain articular cartilage integrity. We have summarized in this review updated findings on OA pathology, related molecular mechanisms, traditional experimental models, and novel OA models represented by human cell-derived microphysiological systems, to inform future basic and translational OA research.

## 2. Risk Factors

**Figure 1:**
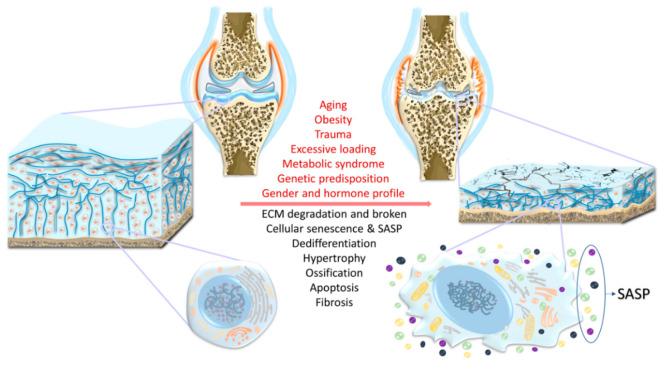
Risk factors, structural alterations, and chondrocyte-specific changes in osteoarthritis (OA).

The known risk factors of OA include aging, obesity, genetic predisposition, acute trauma and chronic overload, gender and hormone profile, and metabolic syndrome [22,23] (Figure 1). However, it should be noted that OA is not the inevitable consequence of these factors. In addition, the different risk factors may act together in the pathogenesis of osteoarthritis. For example, in older adults with anterior cruciate ligament tear, OA develops faster than in younger adults [24].

OA is a multifaceted and heterogeneous disease that affects all joint elements. Compared to the normal joint, OA joint exhibits different clinical and biochemical phenotypes, including breakdown of cartilage, thickening of the subchondral bone, osteophyte and corpus liberum formation, variable degrees of synovial inflammation, narrowed joint space, thickened and fibrotic ligaments, hypertrophy of the joint capsule and, in the knee, damaged menisci. In particular, the number of chondrocytes within cartilage decreases due to increased apoptosis. During OA progression, chondrocytes may undergo dedifferentiation and convert to the hypertrophic and senescent phenotypes. OA chondrocytes also synthesize and secrete SASP, creating a detrimental environment within the joint. Intracellular changes in OA chondrocytes include mitochondrial dysfunction, loss of structure and function of endoplasmic reticulum and Golgi, decreased protein synthesis capacity, as well as increased nuclear size and chromatin shrinkage. ECM: Extracellular matrix; SASP: Senescence-associated secretory phenotype.

### 2.1. Aging

Aging, characterized by the progressive loss of tissue and organ function over time, represents the single greatest risk factor for OA [25]. The Framingham Osteoarthritis Study has established that the frequency of radiographically evident OA, i.e., joint space narrowing, increases with each decade, beginning at 12.9% in people 30–40 years of age and increasing to 43.7% in people over the age of 80 [26]. Several mechanisms of cellular aging have been proposed. One prominent mechanism involves the accumulation of random unrepaired molecular damage to DNA, proteins, and lipids over time that eventually leads to cellular defects and tissue dysfunction, resulting in increased frailty and age-related diseases [27,28]. Sources of this damage are primarily reactive oxygen and nitrogen species produced by mitochondria and cellular stress responses, respectively. The proximal effect of these reactive oxygen species (ROS) is the accumulation of somatic mutations and DNA damage, telomere shortening, protein and lipid damage, and mitochondrial dysfunction. These molecular changes reduce the ability of chondrocytes to main cartilage homeostasis and lower the threshold of damage-inducing load [29]. A wide range of antioxidants have proven effective in reducing induced OA in animal models, including cyclooxygenase-2 (COX-2) inhibitors, hyaluronic acid (HA) and glucosamine, interleukin-1β (IL-1β) receptor antagonists, and polyphenols [30]. As many of the ROS are produced by inefficient mitochondria and radical scavenging, increasing mitochondrial efficiency through the induction of mitophagy (recycling of damaged mitochondria) is the most recent therapeutic target under investigation (Figure 1) [31,32]. Cellular senescence, a phenomenon of irreversible cell growth arrest, is a common cellular outcome of aging (time), and is highly correlated with ROS-induced DNA damage and protein/lipid peroxidation that result from excess ROS (oxidative stress) [33]. Chondrocytes from older adults are shown to exhibit many of the changes typical of cell senescence, such as increased senescence-associated β-galactosidase (SA-β-Gal) activity, enhanced p16 expression, and decreased mean telomere length [34,35]. Interestingly, individuals with OA display a significantly higher number of senescent cells within their cartilage, thus driving elevated expression of detrimental factors that contribute to cartilage degeneration, including IL-1β [35,36], IL-7 [37], and matrix metalloproteinase-13 (MMP-13) [38,39]. However, the exact contribution of aging-associated chondrocyte senescence to OA pathogenesis requires further investigation.

### 2.2. Trauma

Traumatic injury often causes joint instability or intraarticular fractures, causing post-traumatic osteoarthritis (PTOA). Joint injury results in abnormal loading vectors and increased contact stresses that are known to be injurious to articular cartilage [40,41]. PTOA accounts for approximately 12% of all OA [42], and weight-bearing joints are most susceptible. For example, injuries to the knee elements, such as anterior cruciate ligament (ACL) tear and meniscal resection, result in increased radiographic OA occurring at an earlier age [43]. It is estimated that PTOA results in 21% of patients after ACL transection injury, which increases to 48% in those with concomitant meniscal injury [43]. In comparison, 70–80% of radiographic ankle OA cases are of post-traumatic origin, with most patients being younger than those with primary ankle OA [44]. The principal consequences of trauma to the articular cartilage are an immediate loss of cells through necrosis and apoptosis and subsequent increased production of ROS and nitric oxide synthases (NOS) [45]. Early intervention strategies focused on the reduction of cell loss through inhibitors of NOS and apoptosis, and the inhibition of catabolic enzymes such as MMPs and aggrecanase [46]. Newer methods aim to promote anabolic processes through modulation of fibroblast growth factors (FGFs) and WNT signaling and inhibition of hypertrophy [47]. A promising recent reagent is SM04690 (Samumed), an inhibitor of WNT receptor binding that is now in a phase III clinical trial, which has been shown to elicit protective effects on cartilage during joint destruction in an acute cruciate ligament tear and partial medial meniscectomy rodent OA model [48]. Inhibition of β-catenin, an intracellular signal transducer of WNT, by StAx-35R inhibits chondrocyte phenotypic shifting of human OA cartilage explants, and has been reported to result in increased SRY-Box transcription factor 9 (*SOX9*) and aggrecan gene expression and decreased collagen type X α1 chain (*COL10A1*) gene expression [49,50].

### 2.3. Obesity

Obesity, defined as a Body Mass Index (BMI) greater than 30 kg/m^2^, has become a worldwide problem of epidemic proportions. Mechanically, the force exerted on the knee when walking is three to six times one’s body weight, thus a higher body weight significantly increases joint contact stresses [51]. A recent meta-analysis reports an odds ratio (OR) of 1.98 (95% CI 1.57e2.20) and 2.66 (95% CI 2.15e3.28) for developing knee OA in overweight and obese patients, respectively [52]. In contrast, weight loss significantly decreased the risk for the development of knee OA. According to a Framingham study on women, a decrease in body mass index of 2 units or more (weight loss of approximately 5.1 kg) over the 10 years before the designated examination decreased the odds for developing OA by over 50% [53]. In addition to the physical effects, obesity is associated with an increase in systemic metabolic inflammation, and is a risk factor of metabolic syndrome (MS) [54]. Population-based studies conducted in Japan, Nigeria, Egypt, and China have shown that the accumulation of MS components, such as hypertension, dyslipidemia, and impaired glucose tolerance, is strongly correlated with the presence of knee OA and associated advanced radiographic changes, severe pain, and increased functional impairment score [55,56,57,58]. Therefore, the connection between obesity and OA is attributed to not only mechanical loading and “wear-and-tear” at the cartilage surface, but also metabolic and inflammatory mediators, specifically degradative enzymes and adipose tissue-derived cytokines (known as adipokines) [59,60]. Some adipokines, such as leptin, adiponectin, and lipocalin 2, among others, induce production of inflammatory cytokines, including tumor necrosis factor alpha (TNF-α), interleukin (IL)-6, and C-X-C motif chemokine ligand 5 (CXCL5), thus resulting in cartilage matrix damage and subchondral bone remodeling [61]. It is thus not surprising that diets rich in saturated fat have been reported to weaken cartilage metabolism and increase joint damage, leading to OA development. Correlations between high-fat diet and osteocyte changes have also been reported [62].

### 2.4. Chronic Mechanical Overloading/Overuse

Chondrocytes are continuously subjected to physiologic mechanical loading, which is essential for maintaining a homeostatic balance between the catabolic and anabolic processes, mediated via the suppression of proinflammatory cytokines and inflammatory mediators, enhancement of anti-inflammatory signaling, and reduction of the activity of matrix-degrading enzymes [63]. On the other hand, supraphysiological loading has been known to skew this balance towards catabolic processes that lead to cartilage defects, bone marrow lesions, subchondral sclerosis, cartilage thinning, and OA onset [11,64,65,66]. In an ex-elite female athlete study, weight-bearing sports activity was associated with a 2–3-fold increase in radiologic OA risks [67]. Another workload study found that long-term heavy lifting or extended periods of standing at work are associated with hip OA [68]. It should be noted that physiological loading in vivo varies greatly based on anatomical location and history [69], and the area with more cartilage loss is often associated with higher mechanical loading [70]. The chondroprotective effect of physiological mechanical loading is in part achieved through the mechanotransduction mechanisms, such as mechanosensitive ion channels of transient receptor potential vanilloid 4 (TRPV4), and signaling via integrins and primary cilia [69]. For example, under moderate dynamic loading, TRPV4-mediated Ca^2+^ signaling enhances matrix biosynthesis and decreases the expression of catabolic and proinflammatory genes via regulating multiple signaling pathways, including those involving nuclear factor of activated T lymphocytes (NFAT), protein kinase C, NF-κB, JNK1, and cyclic adenosine monophosphate (cAMP) response element-binding protein (CREB) [71]. The signaling pathways involved the degenerative process are, on the other hand, less understood. Available studies show that abnormal mechanical stress may increase the production of proinflammatory mediators, such as prostaglandin E2 (PGE_2_) and nitric oxide (NO), and proinflammatory cytokines, such as IL-1β and TNF-α, that act together to affect cartilage metabolism in multiple ways [65]. Mitogen-activated protein kinase (MAPK), activator protein 1 (AP-1), and NF-κB signaling pathways participate in the upregulation of PGE_2_ and IL-1β-induced NO release [72]. COX-2 could be induced by high intensities of fluid shear stress through the Rac/MEKK1/MKK7/JNK2/c-JunC/EBP pathway [73]. In addition, mechanical injury can cause mitochondrial dysfunction [74], such as the imbalance of superoxide and superoxide dismutase 2 (SOD2), thus resulting in cartilage degeneration [75]. Further research is needed to reveal the molecular mechanisms underlying excessive mechanical loading-induced OA and propose treatment strategies targeting this risk factor.

### 2.5. Genetics

OA is considered a multifactorial polygenic disease that is influenced by multiple genetic and environmental factors [76]. Inheritance studies involving family groups and twin pairs have demonstrated that 39–78% of OA cases can be attributed to genetic factors [77,78]. In addition to directly causing OA, mutations in certain genes could also determine the age of disease onset, sites of affected joints, as well as the severity and progression rate of OA [78]. Most established OA-associated variants are represented by common single-nucleotide polymorphism (SNP) with minor allele frequencies (MAF) > 5% that have moderate to small effect sizes (OR: ~1.1–1.3) [79]. Genome-wide association studies (GWAS) are powerful tools capable of examining hundreds of thousands of SNPs across the genome to establish associations between genetic factors and the risk of complex diseases and traits [80]. By the end of 2019, 90 genome-wide significant OA risk loci have been identified with GWAS, most of which are enriched near genes involved in skeletal development and morphogenesis [81,82]. These include the Wnt pathway genes *HBP1* (HMG-Box transcription factor 1) and *BCL9* (B cell CLL/lymphoma 9 protein), and TGFβ pathway genes *TGFB1*, latent transforming growth factor beta binding protein 1 (LTBP1), *LTBP3*, *SMAD3*, and the recently identified ROCR long non-coding RNA (lncRNA) that acts upstream of *SOX9* during chondrogenic differentiation [83,84,85]. Genetic overlaps with height, hip shape, bone area, and developmental dysplasia of the hip are observed in these sensitive loci, which might alter joint biomechanics and predispose the individual to OA later in life [86,87].

## 3. Regulatory Pathways

**Figure 2:**
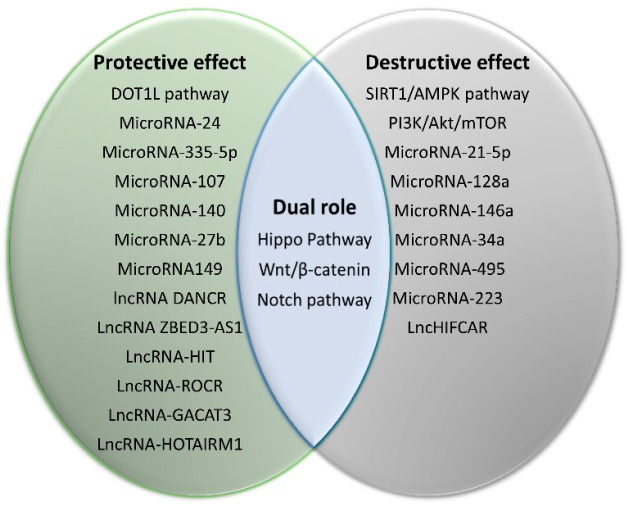
Regulatory factors and pathways involved in OA pathogenesis.

Although how the risk factors may be translated into pathogenic mechanisms is not known, there are a number of signaling and/or regulatory pathways (Table 1) and molecules such as microRNAs and lncRNAs that have been shown to play important roles in articular cartilage mediating the activities of chondrocytes (Figure 2). It should be emphasized that some regulatory pathways have a clearly defined role, either catabolic or anabolic, while others, such as those involving Wnt, may mediate protective or degenerative activities as a function of the state of tissue health.

### 3.1. Wnt/β-Catenin Signaling

Wnts are a family of extracellularly secreted glycoproteins that are involved in numerous biological activities, including cell proliferation, differentiation, polarization, and fate determination; in addition, Wnts have been implicated in the occurrence and development of some diseases via canonical β-catenin-dependent and noncanonical β-catenin-independent signaling pathways [47]. During cartilage development, Wnt/β-catenin signaling activity is strictly regulated in chondrogenesis and chondrocyte maturation [88]. In the adult articular cartilage, excessive Wnt pathway activation under IL-1β stimulation is thought to be an OA progression susceptibility factor and is commonly used to establish an OA model [89]. Increased expression of Wnt pathway activator Wnt1-inducible-signaling pathway protein 1 (WISP-1) is found in both murine and human OA tissues to induce articular cartilage degradation via upregulating the expression of MMPs and aggrecanases in chondrocytes and macrophages [90]. The expression levels of some Wnt pathway antagonists, such as sclerostin, dickkopf WNT signaling pathway inhibitor 1 (DKK1), and secreted frizzled-related protein 3 (sFRP3), may decrease in parallel with OA progression. Upregulating the expression of these antagonists alleviates OA cartilage destruction [91,92,93]. Potential drugs that selectively inhibit the Wnt pathway, such as SM04690, an inhibitor of intranuclear kinases CDC-like kinase 2 (CLK2) and dual-specificity tyrosine phosphorylation-regulated kinase 1A (DYRK1A), or XAV-939, a tankyrase inhibitor, have been shown to be effective in preclinical studies and clinical trials [48,94,95]. However, recent studies revealed a dual role of the Wnt pathway in OA development which deserves attention. Zhu et al. found enhanced articular cartilage destruction in transgenic mice expressing an inhibitor of β-catenin and Tcf in chondrocytes [96]. In the superficial zone of cartilage in adult mice, highly expressed Wnt ligands and stabilized β-catenin upregulate Prg4 expression, which then suppresses cartilage degeneration [97]. In addition, Theologis et al. found that *DKK1* was upregulated in knee synovial fluid (SF) and serum of OA patients, showing positive correlation with OA severity [98]. These results reveal the importance of WNT signaling in maintaining the normal function of chondrocytes and suggest a potential detrimental effect of Wnt inhibitor-based DMOADs on articular cartilage homeostasis and OA progression [96,99]. More research is thus needed to elucidate the role of this pathway. In particular, a better, controlled strategy to fine-tune the spatiotemporal expression of β-catenin is necessary.

### 3.2. PI3K/Akt/mTOR Pathway

The phosphoinositide 3-kinase (PI3K)/protein kinase B (AKT)/mammalian target of rapamycin (mTOR) signaling pathway participates in cell cycle regulation, and is directly related to cellular quiescence, proliferation, cancer, and longevity [100,101]. It also participates in ECM catabolism, anabolism, and chondrocyte homeostasis via regulation of gene expression of MMPs, collagen type II, aggrecan, and a dis-integrin and metalloproteinase with thrombospondin motifs (ADAMTS) [102,103]. A GWAS report found that putative AKT1 rs2498789 and PIK3CA rs7646409 functional variants were associated with knee OA susceptibility in the Chinese Han population [104]. Results from an in vivo study showed that suppression of PI3K/AKT/mTOR signaling pathway promotes chondrocyte autophagy and attenuates the inflammatory response in OA rats [105]. Introduction of pro-autophagic γ-aminobutyric acid receptor-associated protein (GABARAP) to an OA rat model was shown to promote bone marrow mesenchymal stem cell (MSC)-based repair of OA cartilage through the inhibition of PI3K/AKT/mTOR signaling [106]. Several agents targeting the AKT pathway have proven effective in reducing articular cartilage destruction in animal models, providing potential therapeutic agents for the treatment of human OA [107,108,109].

### 3.3. Notch Signaling Pathway

Notch receptors are large single-pass transmembrane proteins that regulate differentiation and apoptosis during embryogenesis and postnatal development through binding to transmembrane ligands expressed on adjacent cells [110,111]. The consequence of Notch signaling can be attributed to different potential Notch receptors, different subcellular locations, and crosstalk between Notch signaling and other signaling pathways [112]. Notch signaling has been clearly shown to play an important role in synovial joint development [113,114,115,116]. In fact, Notch is proposed as a marker of cartilage progenitor cells [117,118]. In the development of mouse limb, intracellular domains of Notch1 and -2 are translocated into the nucleus of chondrocytes to promote their terminal differentiation [118]. However, regarding the exact role of Notch in cartilage hemostasis and OA, apparently contradictory results have been reported in different studies. For example, both Notch signaling activation [118,119] and inhibition [120,121] have been shown to contribute to OA development. However, there is more evidence supporting the pro-degeneration role of Notch signaling in OA pathogenesis. For example, a primary locus in Notch control of cartilage hypertrophy and OA is the transcription factor HES1 (Hes family BHLH transcription factor 1), which appears to act via the intracellular transduction molecule RPBJ (recombination signal binding protein for immunoglobulin kappa J region) to induce common OA-associated genes, including MMP-13, ADAMTS5, and IL-6, among others [110]. Moreover, intraarticular injection of a Notch inhibitor was reported to prevent the development of knee OA in mice [118]. Further studies are needed to comprehensively understand the Notch signaling pathway in the molecular network that regulates cartilage homeostasis and OA pathogenesis.

### 3.4. SIRT1/AMPK Pathway

Sirtuin-1 (SIRT1) and AMP-activated protein kinase (AMPK), two critical sensors that regulate mitochondrial biogenesis and oxidative capacity, have been recognized as therapeutic OA targets [122]. Homocysteine-reduced SIRT1 leads to phosphorylated AMPK and peroxisome proliferator-activated receptor-gamma coactivator (PGC)-1α downregulation, leading to oxidative stress and mitochondrial dysfunction. These homocysteine-induced changes, along with proapoptosis effect, can be reversed by activating SIRT1/AMPK/PGC-1α signaling [123]. Animal studies show that quercetin attenuates oxidative stress-induced apoptosis and mitochondrial dysfunction via upregulated AMPK/SIRT1 signaling pathway in chondrocytes, thus preventing OA progression in murine models [124,125]. In a human study, transcription factor A mitochondrial (TFAM)-mediated activation of the AMPK/SIRT-1/PGC-1α pathway could reverse the deficiency of mitochondrial biogenesis in human OA chondrocytes, suggesting the potential of pharmacologic AMPK activators in mitigating OA progression [126].

### 3.5. Hippo Pathway-YAP/TAZ Signaling

The Hippo signaling pathway is a conserved organ size regulator that acts by controlling cell proliferation and apoptosis [127]. Central to this pathway is a kinase cascade leading from the tumor suppressor Hippo (Macrophage Stimulating 1 (Mst1) and Mst2 in mammals) to the oncoprotein Yki (yes-associated protein 1 (YAP) and tafazzin (TAZ) in mammals) [128]. Although the exact role of the Hippo pathway in cartilage protection and OA development is unclear, the majority of studies show that YAP is a protective effector. For example, YAP was found to cooperate with TEA domain transcriptional factor (TEAD) and activate forkhead box D1 (FOXD1) expression, thus alleviating chondrocyte senescence and OA [129]. In a murine OA model, YAP activation by transgenic overexpression or deletion of the upstream inhibitory kinase Mst1/2 binding sites preserves articular cartilage integrity, whereas downregulation of YAP by inflammatory cytokines through TAK1-mediated phosphorylation promotes cartilage disruption [130,131]. Furthermore, YAP directly interacts with TAK1 and NF-κB signaling by inhibiting substrate TAK1 accessibility and reducing NF-κB-induced matrix-degrading enzyme expression and cartilage degradation during OA pathogenesis [130].

On the other hand, other studies hold that YAP activity increases catabolic gene expression in response to IL-1β. siRNA-mediated suppression of YAP has been shown to inhibit IL-1β stimulated catabolic gene expression, prevent cartilage degradation, and ameliorate OA development. This is supported by the observation in a murine OA model that conditional knock-out (cKO) of YAP preserves collagen type II expression and protects cartilage from degeneration [132]. In fact, intraarticular injection of YAP siRNA was shown to ameliorate OA development in mice [133]. Recently, YAP has also been shown to dictate chondrocyte responses to substrate stiffness. For example, chondrocytes cultured on soft surface display higher collagen type II expression than those on stiff surface, accompanied by lower expression and predominantly cytoplasmic localization of YAP [134]. In addition, knock-out of *YAP* significantly enhances collagen type II expression in chondrocytes seeded on stiff substrate. Finally, YAP is believed to negatively regulate chondrogenic differentiation of MSCs, while chondrogenic signaling de-repression requires *YAP* downregulation [135]. The exact role(s) of YAP in OA initiation and/or progression awaits further investigations.

### 3.6. Disruptor of Telomeric Silencing 1-Like (DOT1L) Pathway

Epigenetic modifications are chemical or physical changes in chromatin that control gene transcription and translation without changing DNA sequence. These modifications include, but are not limited to, DNA methylation, histone modification, chromatin remodeling, and regulatory noncoding RNAs (ncRNAs) [136]. DOT1 is an evolutionarily conserved histone methyltransferase which is involved in epigenetic gene transcription regulation via methylation of lysine-79 of histone H3 (H3K79) [137]. GWAS results have shown that DOT1L safeguards cartilage homeostasis and protects against OA [138]. Maintaining or enhancing DOT1L activity during aging or after trauma might prevent OA onset and progression [139], while DOT1L loss disrupts molecular signature in healthy chondrocytes and increases susceptibility to develop spontaneous and post-traumatic OA in mice [140]. Unexpectedly, the protective function of DOT1L is attributable to inhibition of Wnt signaling by inhibiting the activity of SIRT1 [139], which is generally seen as a protective factor for chondrocytes (see above). More research is needed to verify this finding. In addition to affecting cartilage, DOT1L seems to have an influence on synovial membrane as well. Synovial tissues of OA and RA patients show increased expression of DOT1L at both transcriptional and translational levels, along with the demethylation of its downstream H3K79 target [141]. Given its demonstrated association with OA, epigenetics-based strategies targeting the DOT1L network could be a novel therapeutic option for OA treatment; however, epigenetic modifications are regulated in an extremely complex network and other roles of DOT1L and its targeted genes are largely unknown. Thus, the regulation of DOT1L activity and the functional consequences of manipulation of DOT1L need to be further elucidated before efficient treatments can be developed.

### 3.7. MicroRNAs

An alternative epigenetic mechanism is mediated by microRNAs (miRNAs), short (20–24 nt) non-coding RNAs that regulate gene expression post-transcriptionally by negatively affecting both stability and translation of message RNA (mRNA) via binding to the 3′-untranslated region (3′-UTRs) of specific target genes [142]. Preventing miRNA biogenesis results in skeletal growth defects and premature death, while specific miRNA deletion might be helpful for treating certain diseases [143]. Swingler et al. recently reviewed and summarized the role of RNAs and their targets on chondrogenesis, chondrocyte differentiation, metabolism, apoptosis, senescence, matrix degradation, as well as OA inflammation [144]. Here, we briefly introduce some of these microRNAs. MicroRNA-34a (miR-34a) was the first miRNA linked to chondrocyte apoptosis. miR-34a was upregulated in human OA cartilage, causing OA progression through delta-like protein 1 (DLL1) and PI3K/AKT pathway modulation [145]. miR-34a also induced cell apoptosis via targeting SIRT1, contributing to chondrocyte death. miR-24 suppresses the cell cycle inhibitor P16INK4a, a senescence marker that is increased in OA and in terminal chondrogenesis [146]. miR-495 was overexpressed in human OA cartilage, causing chondrocyte apoptosis and senescence by directly targeting AKT1 and the S6 mTOR system [147]. Other miRNAs related to OA include miR-335-5p [148], miR-107 [149], miR-140-3p [150], miR-223 [151], miR-146^a^ [152], miR-128^a^ [153], miR-27b [154], miR-21-5p [155], and miR-149 [156]. miRNAs clearly play a wide range of important roles in regulating chondrocyte and cartilage hemostasis, but their short half-life, degradation susceptibility, and high mismatch rate limit clinical applications of targeting miRNA [157].

### 3.8. LncRNAs

LncRNAs are defined as long RNA transcripts with lengths exceeding 200 nucleotides that do not encode proteins [158]. LncRNAs have been demonstrated to influence gene expression through transcriptional and translational regulation by recruiting chromatin modification factors, influencing nuclear architecture, acting as decoys or sponges for microRNAs, and modulating the translation and/or stability of mRNAs and proteins [158,159,160,161]. LncRNAs are functionally involved in the entire lifespan of chondrocyte from chondrogenesis to conversion to an OA phenotype. For example, lncRNA differentiation antagonizing non-protein coding RNA (DANCR) regulates both the miR-1305-Smad 4 and miR-216a-5p-JAK2-STAT3 axes, which promote chondrogenic differentiation of human synovium-derived MSCs and stimulate OA chondrocyte proliferation and apoptosis, respectively [162,163]. LncRNA ZBED3-AS1 promotes zbed3 expression, which activates Wnt/β-catenin signaling and promotes chondrogenesis by human synovium-derived MSCs [164]. LncRNA-HIT (HOXA transcript induced by transforming growth factor (TGF)-β) functions in chondrogenesis as an epigenetic regulator through recruitment of the p100/CBP (customs and border protection complex). Suppressing lncRNA-HIT reduces mesenchymal cell condensation and cartilage nodule formation, impairing chondrogenesis in limb bud mesenchyme [165]. LncRNA-ROCR promotes SOX9 expression and chondrogenic differentiation [83]. In terms of disease progression, over 20 different lncRNAs have been identified in regulating ECM degradation, chondrocyte viability, immune response, and angiogenesis that are critical to OA pathogenesis [166]. For example, increased levels of six lncRNAs (HOTAIR (HOX transcript antisense RNA), GAS5 (growth arrest specific 5), PMS2L2 (PMS1 homolog 2 mismatch repair system component pseudogene 2), RP11-445H22.4 (Clone-based (Vega) gene), H19 (H19 imprinted maternally expressed transcript), and CTD-2574D22.4) are associated with the upregulation of MMP-9, MMP-13, and BMP-2 expression in OA cartilage [167]. LncRNA gastric cancer-associated transcript 3 (GACAT3) was highly increased in OA synoviocytes (OAS). Downregulating GACAT3 expression with siRNA arrested cell cycle in G0/G1 phase and increased OAS apoptosis rate, which are mediated by interleukin-6/signal transducer and activator of transcription-3 (IL-6/STAT3) signaling pathway [168]. LncRNA-HOTAIRM1 variant 1 downregulation contributes to OA via regulating the miR-125b/BMPR2 axis and activating the JNK/MAPK/ERK pathway [169]. Because lncHIFCAR is upregulated in OA tissues, lncHIFCAR suppression may improve hypoxia-induced cell injury via positively regulating HIF-1α and HIF-1α target genes [170]. Zinc finger antisense 1 (ZFAS1) expression is downregulated in OA chondrocytes, therefore ZFAS1 overexpression promoted the viability, proliferation, migration, and inhibited OA chondrocyte apoptosis and matrix synthesis by decreasing Wnt3a factors [171]. At present, no therapy targeting lncRNA has been approved by regulatory bodies [172]. More investigations are needed to warrant lncRNA as a potential therapeutic target to treat OA before conducting clinical trials.

The protective effects include anti-inflammation, anti-vascularization, antioxidation, anti-hypertrophy, antiapoptosis, anti-dedifferentiation, and promotion of cartilage formation and proliferation. The destructive effects typically lead to inflammation, hypertrophy, dedifferentiation, accelerating senescence, apoptosis and ossification, and so on. It is noteworthy that some factors and pathways may display protective or destructive functions dependent on different physiological states. AKT: RAC-alpha serine/threonine-protein kinase; AMPK: 5′ AMP-activated protein kinase; DOT1L: Disruptor of telomeric silencing 1-like; mTOR: Mammalian target of rapamycin; PI3K: Phosphatidylinositol 3-kinase; SIRT1: Silent information regulator 1.

## 4. Experimental Models

Experimental models are critical for the study of human diseases. Various in vitro and in vivo models have been established throughout the years to mechanistically understand OA pathologies and develop effective therapies. The translational value of the models is determined by how closely they functionally align with the pathogenesis and progression characteristics of the disease.

### 4.1. In Vivo Models

The OA risk factors discussed above have been recapitulated in different types of animal models (Table 2). No single animal model can mimic all features of human OA and predict all the clinical responses to drugs [173]. Therefore, it is important to note that currently available OA models only cater to a specific mechanism or feature of disease etiology or pathogenesis observed in OA patients. Collectively, the in vivo OA models have significantly advanced our understanding of the disease and its treatment regimen.

#### 4.1.1. Aging-Induced Spontaneous OA Models

Aging is among the highest risk factors for OA. In older adults, OA is the most common cause of limited mobility and compromised quality of life. Spontaneous OA development has been observed in mouse strains including C57/BL6 and STR/Ort mice [174,175]. The time required for mice to develop spontaneous OA phenotypes is much longer than in PTOA models. Wilhelmi et al. [174] reported a high OA incidence of 39–61% in 17-month-old C57/BL6 mice, and only a 19% incidence for those aged 15.5 months. The STR/Ort mice are known to be OA-prone and require a relatively short 12–20-week period to develop OA [175,176]. Articular cartilage degeneration during chronological aging-induced OA development was found to be closely related to the NF-κB signaling pathway in STR/Ort mice [174]. Aging-associated spontaneous OA model has also been established in other species. For example, Dunkin Hartley guinea pigs display higher OA severity with increasing age, reaching moderate to severe OA at 18 months [177]. Spontaneously occurring OA generally appears at a much older age in large animals, such as commercial pig and rhesus macaque [178,179].

#### 4.1.2. Trauma-Induced OA Models

In trauma-induced models, an injurious event, typically instability caused by disrupted joint mechanics, precedes joint arthritis pathogenesis. This trauma can be introduced either invasively or noninvasively. Destabilization of the medial meniscus (DMM) is a well-established and commonly used surgical model where, typically, the medial meniscotibial ligament (MMTL) is transected. As a result, the medial meniscus is displaced medially during activity. This displacement induces abnormal contact stress in the opposing cartilage surfaces which is hypothesized to cause the increase in OA observed clinically after meniscus injury or meniscectomy. The DMM surgery control is usually conducted following the same procedure but without MMTL transection. In the 129/SvEv mouse model, a common background in the production of targeted mutations, mild-to-moderate OA symptoms were observed at 4 weeks post-surgery, and moderate-to-severe OA symptoms were seen at 8 weeks [180]. However, subchondral bone lesions were not observed in this DMM mouse model. Other surgical procedures to induce joint trauma include anterior cruciate ligament transection (ACLT) [181] and partial or total meniscectomy [182]. The ACLT model was the earliest developed OA model that was intended to replicate the degradation of articular cartilage after ACL rupture in humans. However, it is now accepted that this model is unlikely directly comparable to injury in human knee joints [183]. Specifically, immediate and severe joint instability after ACLT leads to the rapid development and progression of OA in animal models, which does not reflect the clinical scenario in human OA [184].

Besides mice and rats, large animals have also been used to generate PTOA models. Using 4-year-old wethers, Cake et al. [185] compared the well-established meniscectomy model with two less traumatic procedures on the meniscus—mid-body transection and cranial pole release—and found that the two new, simpler procedures resulted in similar primary pathological outcomes 3 months post-surgery.

A number of non-surgical PTOA models have been introduced in the past decade, eliminating the confounding effects of invasive injurious procedures [186]. In all these models, the skin or joint capsule of the mice is not disrupted, making the procedure entirely aseptic. In general, noninvasive PTOA models are generated by (1) intraarticular fracture (IAF) of the tibial plateau [186,187], (2) tibia compression of articular cartilage [188,189], or (3) ACL rupture by tibia compression overload [190]. As an example, a custom cradle and an indenter were employed to create closed, intraarticular tibia plateau fracture in the mouse knee, which resulted in OA-like pathological changes in both articular cartilage and subchondral bone [187]. In addition to cartilage degeneration, rapid trabecular bone loss and subsequent partial recovery, as well as considerable bone malformation in the joint space, were also observed following injury.

In previous studies, we reported a portable spring-loaded impactor designed to deliver traumatic, energy-controlled impacts on articular cartilage [191,192]. Using this maneuverable device, we created a first-of-its-kind, injury-induced OA model by subjecting the medial femoral condyle articular cartilage of rabbits to supraphysiological impact. OA characteristics of focal cartilage degeneration, including cell death, tidemark remodeling, loss of cells, and cartilaginous matrix, could be observed for up to 3 months after a single 0.28 J impact was delivered by this impactor [45]. This impact model was later adopted for generating OA-like syndrome in horses [74].

#### 4.1.3. Obesity-Induced OA Models

Higher OA incidence in obese people is attributed to not only excessive joint loading caused by increased body weight, but also the associated systemic inflammation, dysregulated lipid metabolism, and altered adipokine profile [193,194]. Mice fed with a high-fat diet (HFD) exhibit higher levels of proinflammatory cytokines, including IL-1β, IL-6, IL-8, IL-13, leptin, and TNF-α; also present at higher levels in these mice are proteins involved in cartilage metabolism, such as TGF-β1, MMP-13, and vascular endothelial growth factor-α (VEGF-α) [195].

Griffin et al. [196,197] found that HFD (60% kcal from fat, as compared to 10–13.5% from fat for normal diet) caused early-stage knee OA in C57BL/6J mice. Furthermore, researchers observed alleviated OA symptoms in mice that underwent voluntary wheel running, suggesting that higher joint loading per se does not suffice to explain the increased OA incidences in the obesity models [197]. Mice with high-fat-induced obesity have also been used to undergo joint injury, generating PTOA models [198,199]. In addition to accelerated development of age-related spontaneous OA, mice fed with HFD also displayed more severe articular cartilage degeneration in DMM-induced OA as compared with mice fed with lean diet [200]. Underlying the higher OA susceptibility for HFD-fed mice were elevated leptin levels in their plasma and articular cartilage as well as a distinct, longitudinal plasma profile characterized by higher levels of phosphatidylcholines and lysophosphatidylcholines [200]. Schott et al. created a PTOA model in C57BL/6J mice with HFD-induced obesity, and found that the gut microbiome can be targeted to curb systemic inflammation and thus treat OA in obese mice [198]. It should be noted that while previous studies revealed unequivocally that HFD caused exacerbation of PTOA, inconsistent results have been observed in regard to the ability of HFD in OA induction in mouse models [195].

#### 4.1.4. Chemically Induced OA Models

Several chemicals, including monosodium iodoacetate (MIA), papain, collagenase, and quinolone, have been proposed to induce OA in animal models, with MIA being the most commonly used chemical agent to induce OA in mouse and rat models [201,202,203,204]. MIA inhibits glyceraldehyde-3-phosphate dehydrogenase activity, resulting in rapid and widespread chondrocyte death [205]. OA rat models are typically generated by a single intraarticular injection of 100–1200 μg MIA, usually dissolved in physiological saline [201,205,206,207]. Rapid disease progression can be observed after intraarticular MIA injection. Typically, chondrocyte degeneration and necrosis can be observed as early as 1–7 days post-injection, and subchondral bone changes are noted by day 7.

It should be noted that many MIA-induced pathological changes in mice and rates are not typical of human OA. Transcriptional profiling and pathway analysis have revealed little similarities between cartilage tissues from the MIA model of OA in rats and those from human OA joints [201]. Therefore, MIA-induced experimental OA models possess limited clinical relevance and low translatability to human disease. Other OA-inducing chemicals include collagenase and quinolone [203,204]. In general, because of the low clinical relevance of chemical injury-caused pathophysiology, chemically induced models are less popular in OA research.

#### 4.1.5. OA Models Involving Genetic Manipulations

Genetically modified models for OA research are predominantly established in mice because of their short life cycle, high fecundity, breeding efficiency, and being amenable to genetic manipulations [208]. They bear general biological similarities to humans, as reflected in physiology and disease pathogenesis. In particular, the genetic homology between mice and humans presents a useful model to investigate the genetic components of human diseases [209]. A number of genetic modifications have recently been made to target different OA characteristics, such as cartilage matrix degeneration [210], inflammation [211], and chondrocyte hypertrophy and apoptosis [118]. Wang et al. [212] studied mice genetically deficient in complement component 5, and found reduced expression of proinflammatory cytokines and degradative molecules in chondrocytes from joints destabilized by medial meniscectomy, compared to wild type animals. In addition, knock-out of chondrocyte-specific *Epas1*, the gene encoding the signaling molecule hypoxia-inducible factor (HIF)-2α, in mice resulted in inhibition of chondrocyte apoptosis and cartilage destruction in the DMM models of OA [213].

Genetically manipulated animal models produced by gene knock-in and knock-out approaches have been studied to elucidate the protective or destructive role of specific molecules. For example, in *Mmp-13*-knock-out mice, structural cartilage damage was inhibited in surgically induced OA [214]. Neuhold et al. [215] generated *Mmp-13* transgenic mice with cartilage-specific overexpression of *Mmp-13* and observed pathological changes, such as articular cartilage degeneration and synovial hyperplasia, that strongly resemble human OA phenotypes. These loss- and gain-of-function studies clearly indicated the detrimental role of MMP-13 in OA pathogenesis. A small mutation deletion in collagen type II (*Col2a1*) gene was found to result in spontaneous, early-onset articular cartilage degeneration in transgenic *Del1* (+/−) mice [216]. Similarly, *Col9a1*−/−mice, a strain deficient in collagen type IX, experienced faster, spontaneous OA-like changes in the knee joints than their wild type littermates [217]. ADAMTS-4 and ADAMTS-5 have both been recognized as enzymes responsible for aggrecan degradation, a key contributing factor to the degradation of OA cartilage [218]. While knocking out of *Adamts-4* in mice did not reduce aggrecan loss or slow down the progression of surgically induced OA [219], *Adamts-5*-knock-out mice showed significant reduction in cartilage destruction after DMM surgery [220], revealing their different roles in cartilage degeneration. Little et al. [221] generated heterozygous C57BL/6 aggrecan knock-in mice, and this genetic modification protected the mice against cartilage degradation in both PTOA and inflammatory arthritis models through inhibiting aggrecanase-mediated cleavage of aggrecan in the interglobular domain.

Transgenic mice are also used to investigate the roles of different signaling pathways and their associated receptors and ligands in OA development. For example, inhibition of TGF-β signaling by chondrocyte-specific deletion of TGF-β receptor type II (*Tgfβr2*) in mice led to progressive articular cartilage loss and OA-like phenotype, which was ameliorated by further deletion of *Mmp13* or *Adamts5* genes, indicating that they are critical downstream target genes of TGF-β pathway [222]. Indian hedgehog (*Ihh*), the major hedgehog ligand in chondrocytes, was specifically deleted in *Col2a1*-*CreER^T2^ Ihh^fl/f^* mouse cartilage, and the resultant loss in IHH signaling was found to significantly decrease cartilage degeneration in surgically induced OA [223]. Another signaling pathway actively researched in mutant mice is the Wnt pathway due to its critical role in postnatal joint biology and OA development [224,225].

The use of genetically modified mouse strains, typically through the knock-in or -out of known genes, has significantly enhanced our understanding of molecular mechanisms underlying OA pathogenesis. Specifically, these mice are robust tools to study the upstream and downstream network of the target genes, and the mechanistic information facilitates the identification of new disease-modifying targets [226]. Given that OA is a whole joint disease, future studies should focus on mouse models that have been genetically manipulated to target joint tissues other than cartilage, including subchondral bone, synovium, and infrapatellar fat pad (IPFP), to enhance our understanding of the joint pathologies.

### 4.2. In Vitro Models

While biomedical research has been greatly enhanced over the past decades with the use of mammalian animals as a replacement for human subjects, there are also significant limitations and disadvantages, including high costs and social and ethical issues, including the intrinsic genetic differences between human and animals. Thus, in vitro cell and tissue cultures have been used since the development of sterile culture techniques almost a century ago as alternatives. These models are purposed according to the 3R principle—replacing, reducing, and refining animal work—and are implemented to bring about more humane research. We discuss in this section both traditional and novel in vitro OA research models, and their respective advantages and disadvantages (see Table 3).

#### 4.2.1. Monolayer Culture

OA studies involving monolayer culture mostly employ chondrocytes, because degradation of cartilage, in which chondrocyte is the sole cellular component, remains a predominant OA symptom. Monolayer culture serves as a convenient platform to investigate cartilage biology under normal or disturbed conditions. For example, cyclic strain at high magnitude and frequency was found to result in catabolic and degenerative responses by articular chondrocytes [227,228]. To investigate the role of mechanical stress in OA development, porcine chondrocyte monolayer cultures were subjected to cyclic equibiaxial 10% tensile strain (0.5 Hz) [229]. At different times throughout the 24-h period of stretching, chondrocytes showed catabolic responses such as increased expression of *MMPs*, cyclooxygenases, nitrite, and prostaglandin E2. Monolayer cultures of synoviocytes have also been used in OA studies [230,231]. To investigate cartilage–synovium crosstalk, chondrocyte-conditioned medium was used to treat synoviofibroblasts [232]. It was found that a relatively modest chondrocyte-derived IL-6 concentration induced a vastly higher amount of IL-6 secreted by synoviocytes from obese OA patients than from normal-weight patients. This inflammatory response clearly indicated chondrocyte–synoviocyte crosstalk and was further found to be enhanced by leptin, an adipokine related to obesity [232]. Despite the relatively high reproducibility and cost-effectiveness of monolayer cultures, there is growing consensus that a 2-dimensional (2D) plastic surface poorly mimics the in vivo chondrocyte niche. Specifically, in 2D cultures, chondrocytes undergo a dedifferentiation process characterized by the loss of collagen type II and aggrecan expression and increased collagen type I expression [233], thus representing a compromised cellular phenotype. Monolayer cultures have thus gradually fallen out of favor in recent OA studies, and are most often used as a supplement to 3D models.

#### 4.2.2. 3D Engineered Cartilage Tissues

Recognizing that native chondrocytes reside in a 3D microenvironment, researchers have mostly favored 3D cartilage models for in vitro OA studies. High cell density micromass cultures and pellet cultures remain the most commonly used 3D culture approaches for engineering cartilage in vitro. Encapsulating stem cells or primary chondrocytes in biomaterial scaffolds has also been widely employed to create 3D cartilage tissue. In a previous study, dedifferentiated human chondrocytes at passage 5 were redifferentiated under identical conditions in monolayer, pellet cultures, or 3D alginate beads [234]. 3D cultures showed higher chondrogenic potential, while 2D cultures led to hypertrophic and mineralization marker expression. In a similar study, Yeung et al. [235] found that 3D collagen microsphere culture of human OA chondrocytes (hOACs) could better recapitulate the OA phenotypes in vitro, as compared to 2D monolayer culture and traditional 3D pellet culture.

3D engineered cartilage tissues are frequently employed to generate inflammatory OA models. To create an OA model, human articular chondrocytes and mouse RAW 264.7 macrophages were separately encapsulated in 3D poly (ethylene glycol) diacrylate hydrogels and co-cultured in a Transwell system, consisting of a semipermeable membrane to separate the two cell types cultured in the same medium [236]. Through the culture medium shared by the two cell-laden constructs, this model was intended to mimic inflammatory OA features with direct the communication between cartilage and macrophages. In another study, primary human chondrocytes were seeded on silk protein porous scaffolds to engineer 3D cartilage tissues, which displayed OA-like phenotypes when insulted by macrophage-conditioned medium [237].

OA models based on 3D engineered cartilage tissues can also be utilized to test potential OA therapies. For example, an arthritic neocartilage model was generated by challenging human chondrocyte-laden collagen scaffolds with IL-1β and TNF-α. The HA- and platelet-rich plasma used simultaneously rescued the disrupted chondrogenic signaling and enhanced cartilage regeneration [238]. Moreover, the hOAC-laden collagen microspheres, engineered by Yeung et al. [235], responded to OA disease-modifying factors, such as low oxygen tension and TGF-β.

Besides primary chondrocytes, human stem cells also serve as a promising cell source to engineer 3D cartilage tissues [239,240]. In particular, the higher availability of stem cells like adult MSCs makes them preferable in engineering individual-specific cartilage tissues for regenerative medicine and OA studies. It is worth mentioning that the utility of 3D engineered cartilage in OA modeling is strongly dependent on its physiological relevance of biological accuracy. For example, pellet cultures typically require a very large number of cells and cell–ECM interaction is absent until newly formed ECM is generated by cells. On the other hand, in scaffold-based engineered cartilage tissue, cell–cell interaction cannot be achieved initially, due to the physical separation resulting from encapsulation of the cells in the biomaterial scaffold. Of special relevance, it is known that intimate cell–cell interaction, mediated by N-cadherin, a transmembrane protein responsible for homotypic cell–cell adhesion, is critical in initiating mesenchymal chondrogenesis [241]. To overcome these limitations of the pellet and scaffold cultures, we have recently established a development-informed protocol to induce N-cadherin-mediated cellular condensation and subsequent chondrogenesis of human MSCs while encapsulated within their own ECM, which results in robust cartilage formation [242]. In addition, given the requisite crosstalk between cartilage and other joint tissues in the native joint, we have developed an engineered 3D “joint organ” that includes multiple components of the articular joint (see below), which should offer a physiologically more relevant platform for mechanistic studies on joint disorders such as OA.

#### 4.2.3. Tissue Explant Models

Although the exact pathogenesis and etiology of OA are not completely understood, the metabolic state of articular cartilage and its crosstalk with other tissues are believed to play crucial roles in various proposed mechanisms. Cartilage explants from human patients and animal models have thus been widely utilized in mechanistic OA studies. Grenier et al. [243] established an in vitro cartilage degeneration model by treating bovine cartilage explants with collagenase, simulating the matrix damage typically observed in early-stage OA. We have previously created a traumatized OA model by impacting a healthy bovine articular cartilage at 36 MPa [45,191]. In a study to explore the crosstalk between tissue components of the joint organ, particularly subsequent to injurious insults, we examined the interactions between the IPFP and articular cartilage, before and after mechanical trauma. The traumatized cartilage was exposed to IPFP-conditioned culture medium, which was found to aggravate degeneration of the injured cartilage, likely due to elevated IL-6 levels. The traumatized cartilage-conditioned medium also increased IL-6 expression levels in adipocytes and adipose stem cells derived from IPFP, indicating IPFP-cartilage crosstalk [244]. Using IPFP-cartilage and IPFP-meniscus co-cultures, Nishimuta et al. [245] found that co-cultured healthy IPFP could modulate glycosaminoglycan (GAG) metabolism in cartilage and meniscus and stimulate the production and accumulation of GAG in cartilage. Furthermore, the authors labeled newly synthesized sulfated GAGs and proteins with sodium [^35^S]-sulfate and [^3^H]-proline, respectively, in cartilage and meniscus explants and supplemented the culture medium with several adipokines (resistin, leptin, adiponectin, or visfatin) and found that adipokines induced catabolic changes in newly incorporated matrix in both tissues [246]. An in vitro cartilage–synovium explant co-culture model has also been established [247]. The cytokines identified in the co-cultures were found to be more similar to those in OA synovial fluid than those in monocultures of cartilage or synovium. The synovium-secreted factors reduced GAG production in the co-cultured OA cartilage. Interestingly, supplementation with the corticosteroid triamcinolone acetonide (0.1 mM) was found to relieve this inhibitory effect, suggesting the potential utility of such explant systems in screening for OA [247]. The cartilage–synovium explant co-culture model has also been used to investigate the efficacy and mechanisms of potential OA therapies. Using this co-culture model, viscosupplementation (intraarticular injection of HA) was evaluated, and found to benefit OA joints potentially via an anti-inflammatory mechanism of action and a biosynthetic chondroprotective mechanism [248].

#### 4.2.4. Microphysiological Systems

A microphysiological system (MPS), sometimes referred to as an organotypic culture model (OCM), describes an in vitro platform that models human tissues by providing living cells, usually heterogenous in nature, with a microenvironment supporting specific structure and responses that define an organ or tissue. The key features of an MPS include the use of human cells, multiple tissue components, 3D culture, as well as dynamic tissue crosstalk. MPS presents an unprecedented tool for us to mechanistically understand the functions, interactions, and pathogenesis of tissues/organs, and promises to serve as a convenient, versatile component that may be adapted for various drug testing and development scenarios. In view of the “whole joint” nature of OA, an ideal “joint-on-a-chip” should incorporate all the elements of the joint, and ideally is compatible with the application of mechanical load and perturbation.

**Figure 3:**
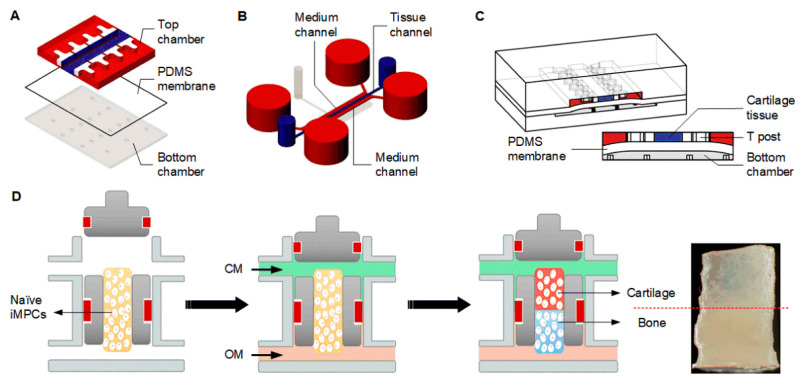
Schematics of cartilage- and osteochondral tissue-on-a-chip microphysiological system (MPS). (**A**–**C**) A cartilage-on-a-chip (COC) system. (**A**) The top and bottom chambers are separated by a polydimethylsiloxane (PDMS) membrane. (**B**) The COC top chamber has a central channel (hosting the 3D microtissues, in blue) and two side channels (for medium supplementation, in red) separated by two rows of T-shaped hanging posts (in white). (**C**) Confined hyperphysiological compression is exerted on the microtissues by pressurizing the bottom actuation compartment of the COC system. (**D**) Schematic of generating the osteochondral tissues-on-a-chip microphysiological system (MPS). After mesenchymal progenitor cells (iMPCs) are encapsulated into a hydrogel scaffold and placed into a dual flow bioreactor, chondrogenic medium (CM) and osteogenic medium (OM) are perfused through the top and bottom flow, respectively, to induce formation of the biphasic osteochondral tissue, with cartilage at the top and bone at the bottom (photographic image of the tissue shown on the right). (Reproduced with permission from Occhetta et al. [249] and Lin et al. [252].)

In reality, the development of MPS for OA studies is at an early stage. In most cases, only cartilage tissue is included. For example, to model excessive mechanical loading-induced OA chondrocyte phenotypes, Occhetta et al. [249] used human articular chondrocyte-laden hydrogels to develop a 3D human cartilage-on-a-chip (COC) constructed on polydimethylsiloxane (PDMS) (Figure 3A,B). Under hyperphysiological confined compression (30% confined compression; Figure 3C), the cartilage microtissues in this microfluidic device displayed increased expression of catabolic enzymes and inflammatory markers, and chondrocyte hypertrophy [249]. Another PDMS-based COC device was designed as an in vitro model of equine OA [250]. The cartilage tissue in this model showed enhanced inflammatory phenotype in response to a 24-h treatment of TNF-α and IL-1β. As noted above, systems for the preparation and culture of osteochondral tissues have been in development for some time, and come in many different configurations and materials. Recognizing the importance of replicating native cartilage–bone crosstalk, we have recently developed microphysiological osteochondral systems derived from human MSCs or iPSCs (Figure 3D) [251,252]. We observed active cartilage–bone crosstalk with IL-1β treatment of either bone or cartilage; we also showed the potential of such osteochondral chips in drug testing and development [252].

**Figure 4:**
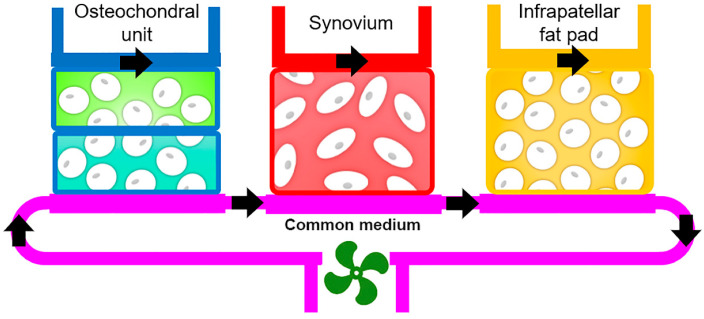
Design of a microphysiological system that simulates the in vivo crosstalk among bone, cartilage, synovium, and infrapatellar fat pad tissues. Each engineered tissue is connected to other tissues through either microfluidics or diffusion, and can thus interact with one another in a real-time manner. The plug-and-play design allows assessment of the contribution of each joint component in the process of OA pathogenesis.

Perhaps the biggest advantage of the MPS platform in OA studies is its capability to enable the interactions and crosstalk among multiple microtissues that correspond to the tissue components present in specific human joints. Initially considered a disease of cartilage degeneration, OA is now recognized as a whole-joint disease, involving and affecting not only bone and cartilage. In particular, synovium and IPFP seem to act as an anatomo-functional unit, which is an emerging idea supported by recent studies [253,254]. Therefore, a human stem cell-derived MPS model, incorporating osteochondral, synovial, and adipose tissues within a chip, has been developed by our group and is coined the “microJoint”, which expands and replicates tissue crosstalk and communication to allow a more complete and holistic understanding of the process of OA pathogenesis (Figure 4).

## 5. Summary and Future Prospects

The significant disease burden of OA and the accompanying compromise to the quality of life of OA patients notwithstanding, development of effective OA prevention and treatment methods have been largely unsuccessful. Current treatments are still limited to lifestyle change, physical therapy, NSAIDs, and end-stage surgical joint replacement. The slow progress in developing novel and effective therapeutic approaches is primarily attributed to our insufficient understanding of OA etiology and pathogenesis [255].

Improved understanding of OA causation and pathogenesis is critical in identifying potential therapeutic targets to prevent disease development and progression. In this review, we have summarized our current understanding of OA pathogenesis. A concise introduction of OA risk factors is provided, as they have been widely discussed and are generally accepted. We have focused on the latest progress in experimental models and regulatory pathways active in OA onset and development. It is clear that interfering with a single known regulatory pathway will be insufficient in preventing or inhibiting OA development. Instead, multiple regulatory pathways must be considered, as well as more upstream targets. A possible strategy is to develop multi-functional molecules that affect several key pathways or activities. For example, the Wnt inhibitors such as XAV939 and lorecivivint, not only suppress inflammatory activities, but also promote chondrogenesis [94,256]. In addition, these agents are known to suppress osteogenesis [239,257], and may thus potentially reduce osteophyte formation.

Valid experimental models of OA are vital to advance research into disease causes and mechanisms, and function as a platform to screen and test potential therapeutics, thus facilitating rational drug design and development. Due to the inherent difference among species in physiology, anatomy, genetics, and metabolism, generating animal models that can faithfully recapitulate all human disease aspects is inherently challenging, which has hindered basic research and translation to clinical application. As discussed above, the integration of multiple 3D engineered joint tissue components into a functionally relevant MPS holds enormous potential as the next generation of OA models. Although establishing human joint-on-a-chip is still at a relatively early stage, promising data have already been obtained, supporting the recapitulation of key physiological and pathological features observed in vivo. As shown in Figure 4, we have recently developed a tissue chip that is capable of mimicking tissue–tissue communication through microfluidic flow or diffusion, which has allowed us to assess the known, important role of joint tissue crosstalk among in OA pathogenesis. Such capability is critical in enabling the refining tissue target(s) in drug development. However, before the complete validation of new OA models, traditional approaches, including animal and 2D cell cultures, will continue to correlate cell and molecular findings with in vivo physiological responses.

There are multiple challenges before MPS becomes an accepted platform for the development of disease modifying OA drugs (DMOADs). In particular, complex mechanics operates in the articular joint, which is not only a part of the native functions of the joint, but also plays critical roles in OA pathogenesis. Although some tissue-on-a-chip models have been developed, how to incorporate the mechanical mechanism in the context of the MPS is not a simple task. In addition, it has been shown that both systemic, in particular, OA-associated pain, and local changes participate in OA progression, and must be incorporated in the MPS. Other requirements include validation of clinical relevance, standardization issues, and regulatory hurdles, but it is highly encouraging that research interest in the tissue/organ-on-a-chip area is rapidly rising.

In conclusion, although there are currently no DMOADs with high efficacy, specificity, potency, and bioavailability [258], with scientific advances in OA pathology and experimental models being continuously made, we are hopefully not far from achieving our goal of finding a cure for this painful and debilitating disease.

## Figures and Tables

**Table 1:** Regulatory pathways mediating chondrocyte functions.

Pathway	Cells Studied	Effects	Ref.
Wnt/β-catenin	Mouse and human OA tissues	Upregulates MMPs and aggrecanases	[90,91,92,93,94]
	Mouse knee chondrocytes	Suppresses cartilage degeneration	[97,98]
PI3K/Akt/mTOR	Rat chondrocytes	Suppresses PI3K/Akt/mTOR promotes cartilage repair and attenuates inflammatory response	[105,106]
Notch	Mouse knee chondrocytes	Induces OA-associated genes and promote OA	[110,118,119]
		Required for articular cartilage and joint maintenance	[120,121]
SIRT1/AMPK	Human and rat knee chondrocytes	Prevents OA progression by attenuating apoptosis and mitochondrial dysfunction	[123,124,125,126]
Hippo/YAP/TAZ	Human and rat knee chondrocytes	Alleviates chondrocyte senescence and reduces matrix-degrading enzyme and cartilage degradation	[129,130]
	Rat knee chondrocytes and human MSCs	Suppresses YAP, preserves collagen type II expression, promotes chondrogenic differentiation of MSCs, and ameliorates OA development	[132,133,134,135]
DOT1L	Mouse and human knee joints and chondrocytes	Prevents OA onset and progression	[139,140,141,142]

**Table 2:** Advantages and limitations of current animal models for studying OA.

In Vivo Models	Advantages	Limitations	Ref.
Aging-induced spontaneous OA models	Simulate natural progression of OA in human Target one of the most important OA risk factors	Need long duration to induce OA High cost due to prolonged housing of animals Sex- and strain-dependent OA incidence	[176,177]
Trauma-induced OA models	Fast OA initiation and development Noninvasive trauma-induced models can be created with precision and minimum infection risk	More severe trauma usually applied than common human knee injuries Much faster and more severe OA induction than in human patients Rely on expertise of surgeon/technician	[186,226]
Obesity-induced OA models	Target a key OA risk factor Replicate both altered joint biomechanics and systemic inflammation seen in obese OA patients	Variability caused by interactions of obesity with genetic and environmental factors	[197,200]
Chemically induced OA models	Ease of OA induction Precise control of chemical dose	Pathogenesis not typical of human OA Low translatability	[204,206]
OA models involving genetic manipulations	Enable studies on the protective/destructive roles of specific genes Facilitate investigations into unknown signaling pathways in OA Can be combined with other models in mechanistic studies	High cost Tend to oversimplify OA pathogenesis Limited clinical relevance of OA induction by a specific gene mutation Most reported genetic manipulations target only cartilage	[212,214]

**Table 3:** Advantages and disadvantages of current in vitro models for OA research.

Current In Vitro Models	Advantages	Limitations	Ref.
Monolayer culture	Support convenient, high-throughput tests High reproducibility	Do not replicate in vivo tissue niche Chondrocytes undergo dedifferentiation	[227,232]
3D engineered cartilage tissues	Create a 3D microenvironment enabling cell–cell and/or cell–matrix interactions Higher chondrogenic potential than 2D culture	Varying biological relevance for different 3D systems Other joint tissues not considered	[235,236]
Tissue explant models	Cells reside in their native environment Study physiology of cartilage as a whole tissue	Properties strongly depend on donor and tissue harvest site Cell death at tissue edges	[245,247]
Microphysiological systems	Support culture of multiple 3D joint tissues to allow their crosstalk Controlled cell culture microenvironment Enable real-time, on-chip analysis Dynamic medium supply supported by microfluidic flow Convenient application of insults and drugs/treatments	Variable biological accuracy due to non-standardized protocols Limited material selection for chip manufacture	[249,252]
